# Psychometric Properties of an Adapted Stigma Scale and Experiences of Stigma Associated with HIV Pre-exposure Prophylaxis Use Among Men Who have Sex with Men: A Mixed Methods Study

**DOI:** 10.1007/s10461-022-03967-0

**Published:** 2023-01-09

**Authors:** David Gillespie, Adam Williams, Fiona Wood, Zoë Couzens, Adam Jones, Richard Ma, Marijn de Bruin, Dyfrig A. Hughes, Kerenza Hood

**Affiliations:** 1 Centre for Trials Research, College of Biomedical & Life Sciences, School of Medicine, Cardiff University, Cardiff, Wales, UK; 2 Division of Population Medicine and PRIME Centre Wales, College of Biomedical & Life Sciences, School of Medicine, Cardiff University, Cardiff, Wales, UK; 3 Public Health Wales NHS Trust, Cardiff, Wales, UK; 4 Policy, Research and International Development, Public Health Wales, Cardiff, Wales, UK; 5 Imperial College London, London, England, UK; 6 Radboud University Medical Center, Institute of Health Sciences, IQ Healthcare, Nijmegen, The Netherlands; 7 Centre for Health Economics and Medicines Evaluation, Bangor University, Bangor, Wales, UK

**Keywords:** HIV, PrEP, MSM, Stigma

## Abstract

Stigma may influence the use of HIV pre-exposure prophylaxis (PrEP). However, there is an absence of robust measures for PrEP-related stigma. We describe an adaptation of a HIV stigma scale for use in PrEP users and experiences of PrEP users in Wales (UK) with regards to PrEP-related stigma. A mixed methods study was conducted where PrEP users completed questionnaire items about PrEP-related stigma and a subset were interviewed about their experiences of taking PrEP. We adapted items from the HIV stigma scale and assessed construct validity and internal consistency. We analysed interview data using a framework approach, with themes focussing on enacted and anticipated stigma in order to identify areas for scale refinement. Our measure had good psychometric properties but additional items may be useful (e.g. specific instances of enacted stigma, concerns around homonegativity). Further work is needed to develop this scale and validate it in a larger sample.

**Supplementary Information:**

The online version contains supplementary material available at 10.1007/s10461-022-03967-0.

## Introduction

The increase in availability of biomedical HIV prevention methods over the past decade, such as pre-exposure prophylaxis (PrEP), has been a significant contributing factor to a global decline in HIV transmission [1]. PrEP involves the use of antiretroviral medication in HIV-negative individuals, and is indicated for individuals whose risk of HIV acquisition is heightened by high-risk sexual behaviour or injecting drug use [2]. Several clinical trials have demonstrated the effectiveness of PrEP in various key populations [3–6], and consequently it is now available in nearly 80 countries worldwide [7]. In Wales, oral tenofovir/emtricitabine (TDF–FTC) has been licensed for use as pre-exposure prophylaxis in people considered to be at risk of HIV acquisition for free through the National Health Service since July 2017 [8]. Its use has been primarily by men who have sex with men (MSM), who make up the majority of HIV diagnoses in Wales. Early work in other settings where PrEP has been introduced highlights the role that stigma may play on the uptake, implementation, and persistence with PrEP. For example, in 2013 Tangmunkongvorakul reported that social stigma may influence adherence to HIV prevention medication and that this could stem from misunderstandings about HIV status and/or being labelled “high sexual risk”. Further work by Peng in 2018 and Edeza in 2021 highlighted stigma as a barrier towards PrEP acceptance [9–11].

Stigma was defined in early work as a distinguishing characteristic that differentiates individuals and serves as a basis for their social devaluation [12]. Subsequent work describes stigma as an umbrella term which describes the co-occurrence of labelling, stereotyping, separation, and status loss or discrimination in a power situation [13, 14]. Building on this, Stangl et al. proposed The Health Stigma and Discrimination Framework, which illustrates stigmatisation processes and how they occur across the socio-ecological spectrum [15]. These later conceptualisations of stigma encourage a transition from single condition, individual-level thinking to a perspective which can encompass many interrelated processes operating at multi-levels which can have an impact on many aspects of an individual’s life.

Stigmatising behaviour towards various groups (e.g. people living with HIV, mental health conditions, intellectual disabilities) has been shown to be associated with poor mental health, a lack of engagement in care, and poor health outcomes [16]. In relation to PrEP, stigmatising behaviour has been reported to typically manifest as misconceptions that the PrEP user is living with HIV (which may trigger HIV-related stigmatising behaviour) or that a PrEP user is sexually irresponsible [9, 17]. Work in Golub describes the former phenomenon as “stigma by association” and how PrEP users are stigmatised as their choice of prevention is seen as “less honourable” than other strategies (e.g. consistent condom use) [18]. Golub proceeds to connect PrEP-stigma with the stigmatisation of sexual desire and expression; an important aspect which positions PrEP-related stigma as a related but distinct concept to HIV-related stigma. Calabrese describes PrEP-related stigma as taking three primary forms: enacted (actual prejudice or discrimination perpetrated by others related to PrEP use), anticipated (expected future prejudice or discrimination perpetrated by others related to PrEP use), and internalised (personal endorsement of prejudice or stereotypes related to PrEP use), and it can operate at intrapersonal, interpersonal, and structural levels [17]. Thus, PrEP-related stigma is a complex multilevel phenomenon that is associated with poorer adherence to PrEP (and hence impacts on its effectiveness in preventing HIV) and requires a multi-faceted approach in order to address it. Indeed, reducing stigma is a key area of focus in the UNAIDS 90:90:90 global AIDS strategy [19].

Recent qualitative work in Wales indicates that stigma, be that HIV- or PrEP-related stigma may play central role in the experience of PrEP use among MSM [20]. The quantitative study of PrEP-related stigma is however limited by the lack of valid and reliable quantitative measures, with several measures currently in development [21, 22]. Existing measures described in the literature are either lacking associated descriptions of their development [23], cover perceptions that people have about others taking PrEP [24, 25], or were not developed in individuals who primarily use PrEP in the UK (i.e. MSM) [26]. Other PrEP-related stigma scales were developed in different contexts (e.g. in Kenya [27] or by recruitment via social media, rather than through locations where PrEP was provided [28]) Few measures based their development on an adaptation of an established HIV-related stigma scale, an area in which there are established measures in use. A recently published systematic review of HIV self-stigma interventions highlighted the common use of the HIV stigma scale developed by Berger et al. [29]. Without good measures available, interventions targeting PrEP-related stigma are unable to reliably measure their success on their direct target outcome and are only able to be inferred through more distal outcomes (e.g. engagement in care).

The aims of this paper are to describe an adaptation of a commonly used HIV stigma scale [29] for use in PrEP users and, by describing the experiences of PrEP users in Wales with regards to different forms and levels of stigma associated with PrEP use, identify areas in which this scale may require further refinement.

## Methods

### Study Design and Participants

We conducted a mixed methods study, comprising an ecological momentary assessment (EMA) study across individuals accessing HIV PrEP through sexual health clinics in Wales and a qualitative semi-structured interview study of a subset of individuals recruited into the EMA study. The study recruited through four of the six health boards offering PrEP services at the time of the study, with one health board declining participation and the other not enrolled due to the dispersed nature of PrEP provision across multiple clinics making participation infeasible. Eligible participants were those prescribed TDF–FTC to prevent HIV-1 and aged at least 16 years. Individuals were excluded if they lacked capacity to consent, were unable to provide a mobile telephone number linked to a smartphone, unable to use the Medication Event Monitoring System (MEMS) cap (an electronic medication bottle cap which recorded the date and time of medication use), or unable to provide an e-mail address. We made no explicit exclusions based on gender, sexual identity, or sexual preference. Participants were approached consecutively during clinic sessions, with 111 individuals approached across 23 clinic sessions.

The study was reviewed and approved by the Wales Research Ethics Committee 3 (reference number: 19/WA/0175) and the analysis of data reported within this article are within the remit of this original approval.

### Procedures

Procedures for each study have been described in full elsewhere [20, 30]. Briefly, 60 individuals recruited into the EMA study were supplied with a MEMS cap to record their medication bottle openings and sent weekly links to an online survey around any condomless sexual intercourse they had engaged in during the previous week. In addition, at dates aligned to clinic visits, participants were asked further questions around health beliefs and behaviours (including stigma), symptoms, and healthcare resource use. This enabled data collection on the date of consent and for three subsequent follow-ups. Data from their clinic notes related to PrEP use and sexually transmitted infection diagnosis and treatment were also extracted. Participants were included in the study for up to 9 months. At the end of their participation in the EMA study, participants were invited to take part in a semi-structured interview about their experiences of taking PrEP. We aimed to interview 20–30 participants, and topic guides were informed by the ABC taxonomy for describing adherence to medication [31] and the theory of planned behaviour [32, 33]. The interview schedule did not cover stigma or discrimination explicitly. However, responses from participants which were related to stigma triggered probes and requests for elaboration from the interviewer. For example:

*Participant*: *I think they might have approached me before [to start taking PrEP], but I think I still had those kind of same anxieties from past experience from the PEP. I wasn’t so much bothered about the label that people would give me if I was on PrEP*.
*Interviewer*: *Okay*
*Participant*: *Because I’ve always seen that it’s a very sensible and clever, well not clever, but like a smart move to kind of make, to help you and other people stay safe and protected. I wasn’t really worried about … yeah …*
*Interviewer*: *So you talked about labels people might give you, on PrEP… what sort of labels, and is that something that you’ve either experienced or you know others have?*

This was pre-planned, as while the primary focus of the interviews was to understand how individuals took PrEP, the research team were aware that stigma may be a topic highlighted by interviewees.

### PrEP-Related Stigma Measure

As part of the EMA study, we adapted items from the HIV stigma scale developed by Berger et al. [29]. While the study of PrEP-related stigma was not the primary goal of the main study, we viewed it an important aspect to measure based on discussions among the research team and stakeholders, in addition to evidence from the literature [11, 17, 23]. Given a competing goal of minimising response burden, we focussed on items related to the *personalized stigma subscale* and *disclosure concerns subscale* as we believed these might directly relate to an individual’s level of adherence to PrEP [34].

Most items were directly adapted from the original scale, replacing HIV status with PrEP use. However, two items were developed to address specific concerns an individual may have with sharing information about their PrEP use with others (misunderstanding around a PrEP user living with HIV and sexual promiscuity). These items were developed in collaboration with the author team and a stakeholder group comprising PrEP users, PrEP providers, and individuals representing HIV and sexual health advocacy and policy. We used 12 items in total, with responses ranging from 1 (“strongly disagree”) to 4 (“strongly agree”) on each. Items were reviewed for content validity and inclusion by mentors and stakeholders (described above) and piloted in sexual health clinics with PrEP users prior to study initiation. Decisions regarding dropping items from the original scale focussed on their perceived relevance and the added burden to participants. See Table 1 for the original items used and how they were adapted to measure PrEP stigma.

**Table 1:** HIV PrEP stigma scale items and their adaptation from the HIV stigma scale

Domain	Original item	Included in adapted version for PrEP?	Adapted item
Personalised stigma	Have lost friends by telling them I have HIV	Y	I have lost friends by telling them that I take PrEP
	Hurt by how people reacted to learning I have HIV	Y	I have been hurt by how people reacted to learning that I take PrEP
	People avoid touching me if they know I have HIV	N	
	Stopped socializing with some due to their reactions	Y	I have stopped socialising with some people due to their reaction when learning that I take PrEP
	People I care about stopped calling after learning	Y	People I care about stopped speaking to me after learning that I take PrEP
	People seem afraid of me because I have HIV	N	
	People have physically backed away from me	N	
	Some people who know have grown more distant	N	
	People who know tend to ignore my good points	N	
	Don’t want me around their children once they know	N	
	I feel set apart, isolated from the rest of the world	N	
	I regret having told some people that I have HIV	Y	I regret having told some people that I take PrEP
	Some fear they’ll be rejected because of my HIV	N	
	Some people act as though it’s my fault I have HIV	N	
	As a rule, telling others has been a mistake	N	
	Some told me HIV is what I deserve for how I lived	N	
	Most with HIV are rejected when others learn	N	
	Knowing, they look for flaws in your character	N	
Disclosure concerns	I never feel I need to hide the fact I have HIV (R)	N	
	I worry people who know I have HIV will tell others	N	
	I am very careful whom I tell that I have HIV	Y	I am very careful whom I tell that I take PrEP
	I work hard to keep my HIV a secret	Y	I work hard to keep my PrEP use a secret
	I told people close to me to keep my HIV a secret	N	
	In many areas of my life, no one knows I have HIV	Y	In many areas of my life, no one knows I take PrEP
	Telling someone I have HIV is risky	Y	Telling someone I take PrEP is risky
	I worry that people may judge me when they learn	N	
	Easier to avoid friendships than worry about telling	N	
	I worry about people discriminating against me	Y	I worry about people discriminating against me because I take PrEP
Additional items included in “anticipated PrEP-related stigma” domain^a^	N/A	N/A	I worry that people will assume that because I take PrEP I have sex with lots of people
	N/A	N/A	I worry that people will assume that because I take PrEP I am HIV positive

^a^Combined with adapted items from “disclosure concerns” domain items of HIV stigma scale

### Data Analysis

Quantitative data analysis first involved assessing construct validity by conducting a confirmatory factor analysis (CFA), whereby the 12 items were hypothesised to conform to a two-factor structure [one related to experiences around personalised stigma related to PrEP use (“Enacted PrEP-related stigma”) and another related to concerns an individual may have around sharing information about their PrEP use with others (“Anticipated PrEP-related stigma”)]. Where participants had not told others that they were taking PrEP (study entry n = 5; follow-up 1 n = 2; follow-up 2 n = 2; follow-up 3 n = 4), the first five stigma items were not asked and instead were imputed as “strongly disagree” for the primary CFA and Cronbach’s alpha calculations and excluded in sensitivity analyses. We used oblique rotation to allow factors to be correlated with each other (as per the original scale) and inspected factor loadings using a Scree plot and eigenvalues (which represent the total amount of variance that can be explained by a given factor). We estimated internal consistency by calculating Cronbach’s alpha [35]. CFA and Cronbach’s alpha values were calculated for each of the four time points (study entry, follow-up 1, follow-up 2, and follow-up 3). Subscale items were summed to provide scores, and items and subscales were described using frequencies with percentages, means with standard deviations, and medians with interquartile ranges as appropriate. We explored the association between PrEP-related stigma and sociodemographics [age, ethnic group (White British/not White British), new/existing PrEP user], sex and relationships [relationship status (single/not single) and number of condomless sexual partners in previous week (0/1/more than 1)], and psychological measures created by the author team and informed by the constructs of the theory of planned behaviour and several proposed extensions (HIV risk perception without PrEP, HIV risk perception with PrEP, injunctive norms around PrEP use, and perceived autonomy around PrEP use) [32, 33] by fitting bivariable two-level mixed effects regression models which accounted for repeated observations within individuals. Two-level logistic regression was fitted to a dichotomised version of the “Enacted PrEP-related stigma” scale (not experienced enacted stigma around PrEP use/has experienced enacted stigma around PrEP use) owing to its skewed nature and two-level linear regression was fitted to the “Anticipated PrEP-related stigma” scale. Data were analysed using Stata v16.1 [36].

Qualitative interviews were audio recorded and transcribed verbatim. We initially analysed our qualitative data relating to stigma using a deductive framework approach [37], whereby data were mapped onto enacted and anticipated stigma (i.e. the focal components of the PrEP stigma scale) at the three main levels at which these operates (intrapersonal, interpersonal, structural) [17]. Our primary aim in our qualitative analysis was to identify areas in which our PrEP-stigma scale may benefit from refinement, and thus we used the “following the thread” approach to integrate our qualitative data with our quantitative data [38]. Coding was led by DG (post-doctoral researcher and CI of the study, with expertise in conducting research around medication use and experienced in conducting and analysing qualitative research), with double coding supported by FW (Professor of Medical Sociology with expertise in conducting and analysing qualitative research) and AW (doctoral student with expertise in Health Psychology experienced in conducting and analysing qualitative research) for consistency and alternative perspectives following the agreement of an initial coding framework. The analysis was supported by the qualitative data analysis software NVivo version 12 [39].

## Results

### Participants

Sixty participants were recruited into the EMA study between September 2019 and January 2020 across four sexual health clinics in Wales. Follow-up concluded in November 2020. All recruited participants were cisgender male, the majority identified as white British ethnicity (53/60, 88.3%), and just under half were educated to degree-level or above (29/60, 48.3%). The majority identified as a gay man (56/60, 93.3%), and all but one participant had sex exclusively with other men. Existing PrEP users made up the majority of recruited participants, with 11 starting PrEP on the day they were recruited (18.3%). At the end of the EMA study, 38 individuals were approached to take part in an interview about their experiences of taking PrEP and 21 were interviewed between May and November 2020. Participants taking part in the interview were broadly similar to those taking part in the wider EMA study. Five of the participants who were interviewed had discontinued PrEP use during the course of the study. Full details of participant characteristics have been reported elsewhere [20, 30].

### Psychometric Properties of PrEP-Related Stigma Measure

Stigma items were available for the majority of participants at each of the time points (60/60 participants at study entry, 57/58 at follow-up 1, 49/54 at follow-up 2, 52/53 at follow-up 3, see Supplementary Table S1 for more detail). One participant did not respond to item 12 (“I worry that people will assume that because I take PrEP I am HIV positive”) at follow-up 2, and one participant did not respond to item 9 (“In many areas of my life, no one knows I take PrEP”) at follow-up 3, from those responding to the follow-up as a whole.

**Fig. 1:**
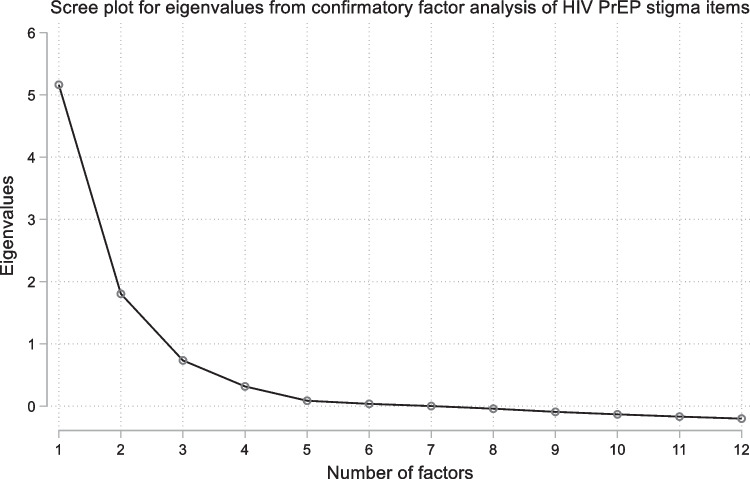
Scree plot for eigenvalues from confirmatory factor analysis of HIV PrEP stigma items. (Eigenvalues represent the total amount of variance that can be explained by a given factor. Eigenvalues greater than 1 explain more variance than an individual item, and hence factors with eigenvalues greater than 1 are indicative of the number of factors which should be considered)

For the CFA at study entry, we found a two-factor solution explained 93% of the total variance, with items loading in the way they did for the HIV stigma scale and thus hypothesised for the present study (i.e. the first five items loading onto factor 1—“Enacted PrEP-related stigma”, and the remaining seven items loading onto factor 2—“Anticipated PrEP-related stigma”). The scree plot in Fig. 1 corroborates this finding, with eigenvalues greater than 1, and hence explaining more variance than an individual item, found for the first two factors. Similar findings were found in a sensitivity analysis where participants who had not responded to the items hypothesised to relate to enacted stigma were excluded (Supplementary Table S2). For the “Enacted PrEP-related stigma” subscale, item total correlations ranged from 0.78 to 0.83 and the Cronbach’s alpha was 0.85. For the “Anticipated PrEP-related stigma” subscale, item total correlations ranged from 0.61 to 0.86 and the Cronbach’s alpha was 0.90. See Table 2 for full details. These findings were replicated across subsequent follow-up time points (see Supplementary Tables S3 and S4 for further details).

**Table 2:** Confirmatory factor analysis for HIV PrEP stigma items and internal consistency of the two HIV PrEP stigma subscales (N = 60)

Subscale	Item	Rotated factor loading	Item-total correlation	Item-rest correlation	Cronbach’s alpha if item removed
Enacted PrEP-related stigma	People I care about stopped speaking to me after learning that I take PrEP	0.759	0.784	0.654	0.815
	I have lost friends by telling them that I take PrEP	0.816	0.829	0.724	0.797
	I have been hurt by how people reacted to learning that I take PrEP	0.701	0.803	0.632	0.832
	I regret having told some people that I take PrEP	0.748	0.794	0.657	0.814
	I have stopped socialising with some people due to their reaction when learning that I take PrEP	0.713	0.778	0.690	0.818
Anticipated PrEP-related stigma	Telling someone I take PrEP is risky	0.790	0.860	0.803	0.869
	I work hard to keep my PrEP use a secret	0.904	0.851	0.803	0.873
	I am very careful whom I tell that I take PrEP	0.839	0.856	0.788	0.869
	In many areas of my life, no one knows I take PrEP	0.826	0.812	0.722	0.878
	I worry about people discriminating against me because I take PrEP	0.721	0.791	0.704	0.880
	I worry that people will assume that because I take PrEP I have sex with lots of people	0.655	0.745	0.641	0.887
	I worry that people will assume that because I take PrEP I am HIV positive	0.496	0.609	0.476	0.905

Based on a two-factor confirmatory factor analysis with oblique rotation to allow for possible correlation between factors

**Fig. 2:**
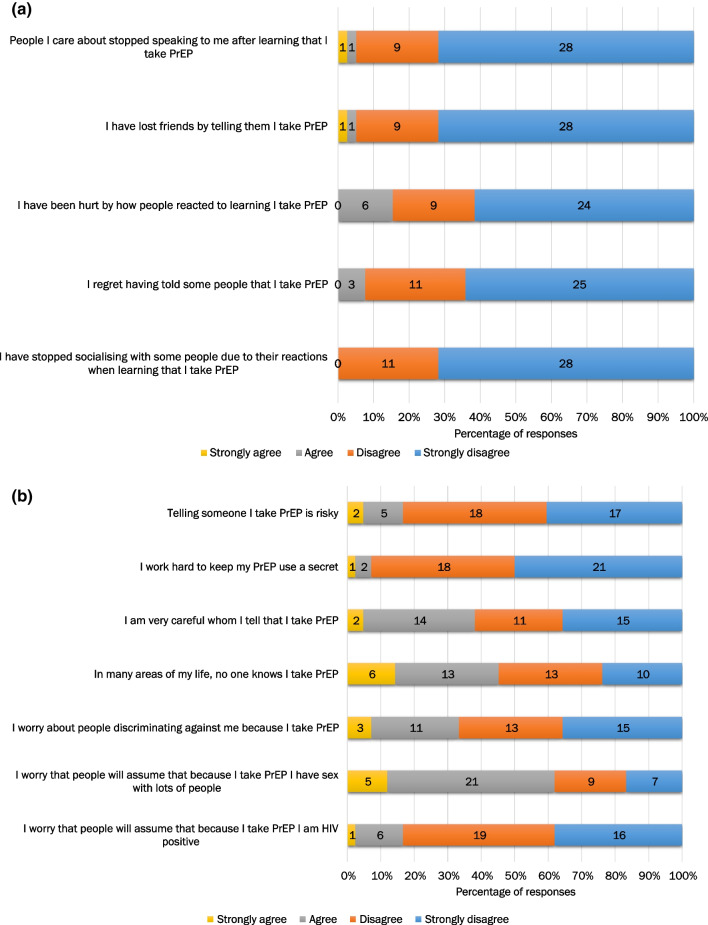
**a** Frequency of responses across enacted PrEP-related stigma items at study entry (N = 55). [Reported by people who had told people that they take PrEP. Numbers in each bar-stack represent the frequency of responses (e.g. 6 individuals responded “agree” to the statement “I have been hurt by how people reacted to learning I take PrEP”)]. **b** Frequency of responses across anticipated PrEP-related stigma items at study entry (N = 60). [Numbers in each bar-stack represent the frequency of responses (e.g. 5 individuals responded “strongly agree” to the statement “I worry that people will assume that because I take PrEP I have sex with lots of people”)]

Summing the Enacted PrEP-related stigma subscale produced scores ranging from 5 (no to low levels of personal experience of PrEP-related stigma) to 20 (high levels of personal experience of PrEP-related stigma), with our sample at study entry ranging from 5 to 13 (median = 5, IQR 5–10) and over half of participants scoring 5 (34/60, 56.7%). Similarly, summing the Anticipated PrEP-related stigma subscale produced scores ranging from 7 (no to low levels of concerns around sharing information about PrEP use with others) to 28 (high levels of concerns around sharing information about PrEP use with others), with our sample at study entry ranging from 7 to 28 (median = 15, IQR 10–18). The correlation between the two PrEP-related stigma subscales was 0.41 (correlation = 0.48 in the sensitivity analysis where individuals who had not responded to the items hypothesised to relate to enacted PrEP-related stigma were excluded). Figure 2a and b illustrate the frequency of different responses across the twelve items at study entry. Scores remained stable over time (Supplementary Tables S3, S4).

### Associations Between PrEP-Related Stigma and Sociodemographic and Psychological Measures

Table 3 displays findings from our analysis of bivariable associations between sociodemographic, sex/relationships, and psychological factors and our PrEP-related stigma measures. For our Enacted PrEP-related stigma measure, we found evidence of an association between a lack of perceived autonomy and experience of enacted stigma (OR 3.53, 95% CI 1.14–10.94, z = 2.18, p = 0.029). For our measure focussing on anticipated stigma, we found evidence of an association between a lack of injunctive norms and higher levels of concerns around sharing PrEP use information with others (β = 0.90, 95% CI 0.24–1.55, z = 2.68, p = 0.007).

**Table 3:** Bivariable associations between sociodemographics, sex/relationships, psychological factors and PrEP-related stigma

Domain	Variable	Level	Enacted PrEP-related stigma (yes/no)				Anticipated PrEP-related stigma (score)			
			OR	95% CI	z**	p	β	95% CI	z*	p
Sociodemographics	Age (years)		1.05	0.97 to 1.13	1.12	0.262	− 0.01	− 0.11 to 0.09	− 0.21	0.837
	Ethnicity	White British	Reference category		− 0.56	0.576	Reference category		− 0.44	0.661
		Not White British	0.50	0.04 to 5.63			− 0.69	− 3.76 to 2.39		
	Used PrEP prior to study	No	Reference category		0.84	0.401	Reference category		− 0.80	0.424
		Yes	2.02	0.39 to 10.45			− 1.18	− 4.09 to 1.72		
Sex and relationships	Relationship status	Single	Reference category		− 0.40	0.692	Reference category		− 1.11	0.267
		Not single	0.71	0.14 to 3.76			− 0.85	− 2.35 to 0.65		
	Number of condomless sexual partners in previous week	Zero	Reference category		4.18	0.124	Reference category		2.64	0.268
		One	0.72	0.19 to 2.66			− 0.59	− 1.43 to 0.25		
		More than one	7.08	0.77 to 64.93			0.17	− 0.78 to 1.12		
Psychological measures	HIV risk without PrEP	No/small chance	Reference category		0.97	0.330	Reference category		1.11	0.267
		Moderate/high chance	1.70	0.59 to 4.91			0.55	− 0.42 to 1.53		
	HIV risk with PrEP	No/small chance	Reference category		− 1.72	0.086	Reference category		− 0.35	0.723
		Moderate/high chance	0.27	0.06 to 1.21			− 0.23	− 1.47 to 1.02		
	People who are important to me approve of me taking PrEP as prescribed (injunctive norms)	Strongly agree	Reference category		1.77	0.077	Reference category		2.68	0.007
		Not strongly agree	2.79	0.90 to 8.66			0.90	0.24 to 1.55		
	Taking PrEP as prescribed is up to me (perceived autonomy)	Strongly agree	Reference category		2.18	0.029	Reference category		0.61	0.543
		Not strongly agree	3.53	1.14 to 10.94			0.29	− 0.63 to 1.21		

*Analysis based on up to 218 observations within 60 participants (minimum = 204 within 58 participants). β coefficient represents mean difference or mean per unit increase (for age)

**Where a joint test of a variable with greater than two categories (and number of categories = k) is presented, the test statistic is a χ^2^ value with k − 1 degrees of freedom

### Qualitative Analysis Highlighting Areas for Scale Development

Participants who were interviewed in the sub study were similar in terms of their level of reported stigma compared to those not interviewed. For example, at study entry the median Enacted PrEP-related stigma score was 5 (IQR 5–10) and Anticipated PrEP-related stigma score was 15 (IQR 10–18) for those who were interviewed. These scores were comparable for those who were approached and not interviewed [medians (IQRs) = 5 (5–7) and 14 (11–17) respectively] and those who were not approached for interview [medians (IQRs) = 5.5 (5–10) and 16 (10–18) respectively].

Within the enacted stigma subscale, we identified substantial floor effects. This is in spite of interview data highlighting specific experiences of enacted stigma at interpersonal- and structural-levels. At the interpersonal-level, participants described instances where their friends had made negative comments about PrEP and its association with deviancy.

You have a couple of people that would say, “Oh I don’t agree with that” and it was often because … it’s a way of fuelling a deviant sexual life. But I would say nine out of ten of the responses were always positive, and of those one out of ten, there was never anything particularly against me personally, it was just around the subject in general. [PID 8, aged 20 to 30 years, discontinued PrEP]

Furthermore, participants recounted experiences where their partner had questioned the purpose of their PrEP use.

He (Partner) said to me, “When are you Going to meet with Anybody?” So I said, “I don’t Really feel like it.” And then he said, “Well is there any Point in you Still Taking PrEP, Because you’re only ever with me?” It wasn’t a case of he doesn’t Support me. He was just sort of Saying, well, you don’t meet with Anybody Except me. I haven’t got Anything. [PID 4: aged 31+ years, continued PrEP]

At a structural level, participants described instances where a lack of awareness of understanding about PrEP in healthcare professionals who worked outside of sexual health led to assumptions that they were living with HIV, rather than taking antiretrovirals for prevention.

A couple of weeks ago, I had a call off my nurse [non-sexual health specialist for healthcare need unrelated to PrEP] and I told her that I was on PrEP, and she turned round, and just said “Oh that’s okay then. I just didn’t realise”. And then I had this feeling that [she thought] I was already HIV. So I mentioned it and she said “Oh right, I’m so sorry”. [PID 11, aged 31+ years, continued PrEP]

While scores demonstrated better distributional properties for the anticipated stigma subscale, additional items regarding implied sexual orientation may had led to further improvement.

It [taking PrEP to prevent HIV] tells people [about] my sexual activity…. So again I think it’s … wanting to be discreet about … the kind of sexual activity I have. Fundamentally I’m gay but I’m not out… So I don’t want that to be common knowledge. [PID 31, aged 31+ years, continued PrEP]

This discretion extended from the interpersonal- to structural-level, with concerns raised about interacting with healthcare professionals and sharing details about PrEP use in one-to-one and more open environments.

Originally when I was prescribed it at the clinic I did ask for them not to even notify my doctor that I was taking it. Last week I had to have some antibiotics, and I had to tell them the GP that I was taking it [PrEP] because obviously I wasn’t sure whether the antibiotics contradicted the PrEP… His suggestion was to tell the pharmacy and I mean the quite difficult bit for me was having to say that I was taking PrEP to a pharmacist in the middle of the pharmacy. And then he says to me, I don’t know what you’re talking about, what PrEP are you talking about? So it was like how do I get around this in an open environment? … I live in an area where everything is quite communal. So you got to the chemist, okay you might not know the pharmacist himself, but it’s that you know the staff the other side of the desk or whatever, so, you know although there is a level of confidentiality it always gives you that doubt of actually what they’re sharing with friends and family or whatever. [PID 31, aged 31+ years, continued PrEP]

## Discussion

In this observational study of HIV PrEP users in Wales, we investigated the psychometric properties of an adapted version of the HIV stigma scale by Berger et al. that aimed to measure personal experiences individuals had of stigmatising behaviour associated with their PrEP use and concerns they had about telling other people that they take PrEP (the PrEP-related stigma scale). We also interviewed a subset of study participants and analysed their data focussing on areas for scale refinement. We found that the adapted subscales demonstrated good construct validity and internal consistency. For the enacted stigma subscale, there was some evidence of floor effects, with over half of participants indicating no to very low levels of personal experience of stigmatising behaviour around their PrEP use. Nevertheless, we found evidence of a moderate correlation between both subscale scores and also associations between a lack of perceived autonomy around PrEP use and enacted PrEP-related stigma as well as a lack of injunctive norms and higher levels of anticipated stigma. Qualitative analysis highlighted areas for scale refinement. For example, by providing greater specificity when asking about enacted stigma (e.g. items asking whether individuals have been judged about the implied type of sexual activity they engage in because they are a PrEP user), within-partner stigmatising behaviour, and misconceptions that the PrEP user was living with HIV. Data related to anticipated stigma were mostly consistent with the items captured by our corresponding subscale. However, key aspects not captured by our subscale were the implied sexual orientation of a PrEP user and level of discretion desired around this—particularly when consulting healthcare professionals outside of sexual health. Thus, our work adds a PrEP-related stigma scale to the literature, with good psychometric properties within this initial sample, and identifies areas in which it can be further refined to better capture both enacted and anticipated stigma related to PrEP use.

This is one of a few studies that has attempted to develop a specific stigma measure for PrEP users. Work by Siegler et al. describes the validity of a brief HIV PrEP stigma scale developed among MSM recruited via social media [28]. Their work differs from ours in terms of their sampling method (PrEP users based in the USA identified through social media compared to PrEP users obtaining PrEP through clinics in Wales, UK), and development strategy (adaptation across a range of measures compared to adaptation from a single measure). Both scales demonstrate good psychometric properties but require further validation in larger samples of more diverse PrEP users. A recent study adapting a shortened version of the HIV stigma scale for PrEP-eligible individuals in Kenya was published by Atkins et al. [27]. This adapted scale similarly demonstrated good psychometric properties. We conducted this study by recruiting a nationally representative sample of individuals accessing PrEP through NHS sexual health clinics in Wales, with no evidence of systematic bias between the full sample and the subset interviewed [20, 30]. Our mixed methods approach allowed us to explore the psychometric properties of our adapted scale while determining within the same sample whether additional items might have been important. While participants completed items related to stigma during the quantitative study, qualitative data related to stigma were generated organically, rather than through targeted questions. While this meant that not all participants discussed aspects associated with stigma during their interviews, the data that were captured are likely to reflect key points that the participant deemed to be associated with their PrEP use, rather than data generated following a priming question which might have encouraged participants to search for an answer regardless of its level of relevance.

A limitation to this work is its relatively small sample size. These findings would need confirming in a larger study. Furthermore, while nationally representative, our sample were primarily white British MSM taking PrEP according to a daily regimen. The validity of this PrEP-related stigma scale, in addition to experiences of different forms and levels of stigma experienced by non-White, non-British, non-MSM may vary and should be confirmed. PrEP users following an alternative dosing regimen or formulation (e.g. event-based dosing, injectable PrEP) may similarly experience different forms of PrEP-related stigma. For example, by adopting a non-daily dosing regimen (e.g. event-based or on-demand PrEP), there may be a reduction in stigma associated with a misconception about an individual’s HIV status. PrEP-related stigma in individuals taking event-based PrEP and other non-daily PrEP regimens (e.g. injectable PrEP) require investigating in further work. In particular, whether PrEP-related stigma measures require further refinement, the extent to which stigma influences (or would influence) an individual’s decision to adopt and continue with a particular PrEP regimen, and whether stigma played a role in switching between regimens.

It is also possible that those who had already experienced high levels of stigma related to their PrEP use declined participation in the quantitative study (which involved the collection of electronically monitored PrEP use data) or qualitative sub-study (though we found negligible differences between those included in the quantitative study who were and were not interviewed).

Our qualitative findings, while informative for improving the validity of our scale, largely reflect those found in other settings. Indeed, work by Brooks et al. studying experiences of anticipated and enacted PrEP stigma among Latino MSM in Los Angeles identified themes related to the nature of a PrEP user’s sexual behaviour, PrEP-induced conflict in relationships, and perceptions that PrEP users are actually living with HIV [40]. Discussing PrEP use outside of sexual relationships was a topic highlighted directly and indirectly in our qualitative and quantitative work respectively. This may reflect social norms related to when it is deemed appropriate to talk about sex, as highlighted from work conducted with PrEP users in Antwerp and Amsterdam [41]. The implicit assumption that a PrEP user is gay, and the concerns associated with this assumption, is a finding highlighted in a meta-ethnography by Edeza et al. [9].

People who take PrEP are subjected to various forms of stigmatising behaviour associated with their PrEP use. Further work is needed to develop, evaluate, and implement interventions to combat stigma. These could take various forms, depending on their intended target. In a review by Curley et al., PrEP was found to increase sexual satisfaction, pleasure, quality, and emotional intimacy [42]. Furthermore, PrEP enabled an increase in sexual options and opportunities. These are important outcomes that indicate the role that PrEP can play in enhancing sexual wellbeing [43], with the sexual freedom coupled with reduced fear and anxiety encouraging a reframing of the word “promiscuous” from having negative connotations to becoming an affirmative word.

This work also highlights the tension between HIV- and PrEP-related stigma. The perceived stigma associated with HIV appears to be a key motivator for some individuals accessing PrEP, with conversations around PrEP seen to enable wider discussion about current HIV prevention, treatment, and prognosis information. While this latter aspect provides a useful opportunity to dispel any HIV-related stigmatising views among PrEP users, the former aspect emphasises the care that must be taken when discussing PrEP options with potential users (i.e. so as to not reinforce any stigmatising views around HIV).

Stigma is difficult to measure and may vary across different demographic groups/subgroups and even within the same populations over time. Furthermore, some groups may also be more impacted by intersectional stigma than others [44]. Our sample included primarily white British MSM, who themselves may experience intersectional stigma as a result of their sexual orientation. Further work is needed to consider the impact of intersectional stigma, stigma in different demographic groups/subgroups, and the extent to which stigma components change over time (e.g. as awareness of PrEP increases).

As PrEP uptake increases, this may change societies understanding of “safe sex” within the context of HIV. Indeed, work by Haire et al. highlights the decreasing centrality of condoms in HIV-risk reduction and how stigma may shift to those who are not taking PrEP as PrEP becomes accepted as a positive risk reduction method [45].

## Conclusion

We found preliminary evidence that an adapted scale aiming to measure PrEP-related stigma has good psychometric properties within a sample of White MSM PrEP users. Our findings also suggest additional items that may be relevant to capture stigma, such as specific instances of enacted stigma and concerns around the implied sexual orientation of the PrEP user. Further work is needed to develop this scale, validate it in a larger sample, and determine the extent to which it remains a valid measure of PrEP-related stigma in different demographic groups and remains valid over time, as PrEP use becomes more widespread and varied in its formulations.

## Supplementary Information

Below is the link to the electronic supplementary material.

Supplementary file1 (DOCX 16 kb)

## Data Availability

Quantitative data are available from the corresponding author upon request and subject to a data sharing agreement. Qualitative data (thematic coding matrices for all developed themes) are available on request from the authors. Due to privacy/ethical restrictions, other data (e.g. full transcripts) are not available.

## References

[1] Bavinton BR, Grulich AE (2021). HIV pre-exposure prophylaxis: scaling up for impact now and in the future. Lancet Public Health.

[2] World Health Organization. HIV/AIDS: pre-exposure prophylaxis. [cited 2021 May 9]. Available from: https://www.who.int/hiv/topics/prep/en/.

[3] Grant RM, Lama JR, Anderson PL, McMahan V, Liu AY, Vargas L (2010). Preexposure chemoprophylaxis for HIV prevention in men who have sex with men. N Engl J Med.

[4] Choopanya K, Martin M, Suntharasamai P, Sangkum U, Mock PA, Leethochawalit M (2013). Antiretroviral prophylaxis for HIV infection in injecting drug users in Bangkok, Thailand (the Bangkok Tenofovir Study): a randomised, double-blind, placebo-controlled phase 3 trial. Lancet.

[5] McCormack S, Dunn DT, Desai M, Dolling DI, Gafos M, Gilson R (2016). Pre-exposure prophylaxis to prevent the acquisition of HIV-1 infection (PROUD): effectiveness results from the pilot phase of a pragmatic open-label randomised trial. Lancet.

[6] Molina J-M, Charreau I, Spire B, Cotte L, Chas J, Capitant C (2017). Efficacy, safety, and effect on sexual behaviour of on-demand pre-exposure prophylaxis for HIV in men who have sex with men: an observational cohort study. Lancet HIV.

[7] Schaefer R, Schmidt HMA, Ravasi G, Mozalevskis A, Rewari BB, Lule F (2021). Adoption of guidelines on and use of oral pre-exposure prophylaxis: a global summary and forecasting study. Lancet HIV.

[8] Gething V. Written statement: availability of pre-exposure prophylaxis (PrEP) to prevent HIV. Welsh Gov. 2020 [cited 2021 May 9]. Available from: https://gov.wales/written-statement-availability-pre-exposure-prophylaxis-prep-prevent-hiv.

[9] Edeza A, Karina Santamaria E, Valente PK, Gomez A, Ogunbajo A, Biello K (2021). Experienced barriers to adherence to pre-exposure prophylaxis for HIV prevention among MSM: a systematic review and meta-ethnography of qualitative studies. AIDS Care.

[10] Peng P, Su S, Fairley CK, Chu M, Jiang S, Zhuang X (2018). A global estimate of the acceptability of pre-exposure prophylaxis for HIV among men who have sex with men: a systematic review and meta-analysis. AIDS Behav.

[11] Tangmunkongvorakul A, Chariyalertsak S, Rivet Amico K, Saokhieo P, Wannalak V, Sangangamsakun T (2013). Facilitators and barriers to medication adherence in an HIV prevention study among men who have sex with men in the iPrEx study in Chiang Mai, Thailand. AIDS Care.

[12] Major B, O’Brien LT (2005). The social psychology of stigma. Annu Rev Psychol.

[13] Hatzenbuehler ML, Phelan JC, Link BG (2013). Stigma as a fundamental cause of population health inequalities. Am J Public Health.

[14] Link B, Hatzenbuehler ML (2016). Stigma as an unrecognized determinant of population health: research and policy implications. J Health Polit Policy Law.

[15] Stangl AL, Earnshaw VA, Logie CH, Van Brakel W, Simbayi LC, Barré I (2019). The health stigma and discrimination framework: a global, crosscutting framework to inform research, intervention development, and policy on health-related stigmas. BMC Med.

[16] Rao D, Elshafei A, Nguyen M, Hatzenbuehler ML, Frey S, Go VF (2019). A systematic review of multi-level stigma interventions: state of the science and future directions. BMC Med.

[17] Calabrese SK (2020). Understanding, contextualizing, and addressing PrEP stigma to enhance PrEP implementation. Curr HIV/AIDS Rep.

[18] Golub SA (2018). PrEP stigma: implicit and explicit drivers of disparity. Curr HIV/AIDS Rep.

[19] UNAIDS. Global aids strategy 2021–2026 end inequalities. End Aids. UNAIDS. 2021; p. 160. Available from: https://www.unaids.org/en/Global-AIDS-Strategy-2021-2026.

[20] Gillespie D, Wood F, Williams A, Ma R, de Bruin M, Hughes DA, Jones AT, Couzens Z, Hood K. Experiences of men who have sex with men when initiating, implementing and persisting with HIV pre-exposure prophylaxis. Health Expect. 2022;25(4):1332–41. 10.1111/hex.13446 PMC9327834 35426223

[21] Klein H, Washington TA (2019). The pre-exposure prophylaxis (PrEP) stigma scale: preliminary findings from a pilot study. Int Public Health J.

[22] Calabrese SK, Dovidio JF, Tekeste M, Taggart T, Galvao RW, Safon CB (2018). HIV pre-exposure prophylaxis stigma as a multidimensional barrier to uptake among women who attend planned parenthood. J Acquir Immune Defic Syndr.

[23] Golub SA, Gamarel KE, Rendina HJ, Surace A, Lelutiu-Weinberger CL (2013). From efficacy to effectiveness: facilitators and barriers to PrEP acceptability and motivations for adherence among MSM and transgender women in New York City. AIDS Patient Care STDS.

[24] Mustanski B, Ryan DT, Hayford C, Phillips G, Newcomb ME, Smith JD (2018). Geographic and individual associations with PrEP stigma: results from the RADAR cohort of diverse young men who have sex with men and transgender women. AIDS Behav.

[25] Algarin AB, Hee Shrader C, Hackworth BT, Varas-Diaz N, Fennie KP, Sheehan DM (2021). Development and validation of the Community PrEP-Related Stigma Scale (Community-PSS). AIDS Educ Prev.

[26] Velloza J, Hosek S, Donnell D, Anderson PL, Chirenje M, Mgodi N (2021). Assessing longitudinal patterns of depressive symptoms and the influence of symptom trajectories on HIV pre-exposure prophylaxis adherence among adolescent girls in the HPTN 082 randomized controlled trial. J Int AIDS Soc.

[27] Atkins K, Kan L, Musau A, Reed J, Were D, Mohan D. Adaptation and psychometric evaluation of a scale to measure oral pre-exposure prophylaxis-related stigma among key and vulnerable populations in Kenya. J Int AIDS Soc. 2022;25:e25929. 10.1002/jia2.25929 PMC9274213 35818870

[28] Siegler AJ, Wiatrek S, Mouhanna F, Amico KR, Dominguez K, Jones J (2020). Validation of the HIV pre-exposure prophylaxis stigma scale: performance of likert and semantic differential scale versions. AIDS Behav.

[29] Berger BE, Ferrans CE, Lashley FR (2001). Measuring stigma in people with HIV: psychometric assessment of the HIV stigma scale. Res Nurs Health.

[30] Gillespie D, Couzens Z, de Bruin M, Hughes DA, Jones A, Ma R (2022). PrEP use, sexual behaviour, and PrEP adherence among men who have sex with men living in Wales prior to and during the COVID-19 pandemic. AIDS Behav.

[31] Vrijens B, De Geest S, Hughes DA, Przemyslaw K, Demonceau J, Ruppar T (2012). A new taxonomy for describing and defining adherence to medications. Br J Clin Pharmacol.

[32] Ajzen I (1991). The theory of planned behavior. Organ Behav Hum Decis Process.

[33] Banas K, Lyimo RA, Hospers HJ, van der Ven A, de Bruin M (2017). Predicting adherence to combination antiretroviral therapy for HIV in Tanzania: a test of an extended theory of planned behaviour model. Psychol Health.

[34] Arnold T, Brinkley-Rubinstein L, Chan PA, Perez-Brumer A, Bologna ES, Beauchamps L (2017). Social, structural, behavioral and clinical factors influencing retention in pre-exposure prophylaxis (PrEP) care in Mississippi. PLoS ONE.

[35] Boateng GO, Neilands TB, Frongillo EA, Melgar-Quiñonez HR, Young SL (2018). Best practices for developing and validating scales for health, social, and behavioral research: a primer. Front Public Health.

[36] StataCorp (2019). Stata statistical software: release 16.

[37] Gale NK, Heath G, Cameron E, Rashid S, Redwood S (2013). Using the framework method for the analysis of qualitative data in multi-disciplinary health research. BMC Med Res Methodol.

[38] O’Cathain A, Murphy E, Nicholl J (2010). Three techniques for integrating data in mixed methods studies. BMJ.

[39] QSR International Pty Ltd. NVivo [Internet]. 2018. Available from: https://www.qsrinternational.com/nvivo-qualitative-data-analysis-software/home.

[40] Brooks RA, Landrian A, Nieto O, Fehrenbacher A (2019). Experiences of anticipated and enacted pre-exposure prophylaxis (PrEP) stigma among Latino MSM in Los Angeles. AIDS Behav.

[41] Reyniers T, Zimmermann HML, Davidovich U, Vuylsteke B, Laga M, Hoornenborg E (2021). The social meanings of PrEP use—a mixed-method study of PrEP use disclosure in Antwerp and Amsterdam. Sociol Health Illn.

[42] Curley CM, Rosen AO, Mistler CB, Eaton LA (2022). Pleasure and PrEP: a systematic review of studies examining pleasure, sexual satisfaction, and PrEP. J Sex Res.

[43] Mitchell KR, Lewis R, O’Sullivan LF, Fortenberry JD (2021). What is sexual wellbeing and why does it matter for public health?. Lancet Public Health.

[44] Abubakari GMR, Dada D, Nur J, Turner D, Otchere A, Tanis L (2021). Intersectional stigma and its impact on HIV prevention and care among MSM and WSW in sub-saharan african countries: a protocol for a scoping review. BMJ Open.

[45] Haire B, Murphy D, Maher L, Zablotska-Manos I, Vaccher S, Kaldor J (2021). What does PrEP mean for “safe sex” norms? A qualitative study. PLoS ONE.

