# Supplementary material for: Psychometric Properties of an Adapted Stigma Scale and Experiences of Stigma Associated with HIV Pre-exposure Prophylaxis Use Among Men Who have Sex with Men: A Mixed Methods Study
Source: AIDS Behav. 2023 Jan 9;27(7):2397–410. doi: 10.1007/s10461-022-03967-0 (PMC10224859; doi:10.1007/s10461-022-03967-0)
Supplement: Supplementary file 1 — Supplementary file1 (DOCX 16 kb) [file 10461_2022_3967_MOESM1_ESM.docx]

**Psychometric properties of an adapted stigma scale and experiences of stigma associated with HIV pre-exposure prophylaxis use among men who have sex with men: a mixed methods study [Supplementary material]**

**Supplementary Table S1:** Stigma item data availability across time

|  | | **Study entry** | **Follow-up 1** | **Follow-up 2** | **Follow-up 3** |
| --- | --- | --- | --- | --- | --- |
| **Active participants*** | | 60 | 58 | 54 | 53 |
| **Provided data for data collection wave**** | | 60 | 57 | 49 | 52 |
| Enacted PrEP-related stigma*** | People I care about stopped speaking to me after learning that I take PrEP | 60 | 57 | 49 | 52 |
|  | I have lost friends by telling them that I take PrEP | 60 | 57 | 49 | 52 |
|  | I have been hurt by how people reacted to learning that I take PrEP | 60 | 57 | 49 | 52 |
|  | I regret having told some people that I take PrEP | 60 | 57 | 49 | 52 |
|  | I have stopped socialising with some people due to their reaction when learning that I take PrEP | 60 | 57 | 49 | 52 |
| Anticipated PrEP-related stigma | Telling someone I take PrEP is risky | 60 | 57 | 49 | 52 |
|  | I work hard to keep my PrEP use a secret | 60 | 57 | 49 | 52 |
|  | I am very careful whom I tell that I take PrEP | 60 | 57 | 49 | 52 |
|  | In many areas of my life, no one knows I take PrEP | 60 | 57 | 49 | 51 |
|  | I worry about people discriminating against me because I take PrEP | 60 | 57 | 49 | 52 |
|  | I worry that people will assume that because I take PrEP I have sex with lots of people | 60 | 57 | 49 | 52 |
|  | I worry that people will assume that because I take PrEP I am HIV positive | 60 | 57 | 48 | 52 |

*Recruited and not withdrawn. **Provided at least one response at time point. ***Participants did not respond to these questions if they had not told others about their PrEP use. Their data have been imputed to strongly disagree (study entry n = 5; follow-up 1 n = 2; follow-up 2 n = 2; follow-up 3 n = 4).

**Supplementary Table S2:** Confirmatory factor analysis for HIV PrEP stigma items and internal consistency of the two HIV PrEP stigma subscales (sensitivity analysis, N = 55)*

| **Sub-scale** | **Item** | **Rotated factor loading** | **Item-total correlation** | **Item-rest correlation** | **Cronbach’s alpha if item removed** |
| --- | --- | --- | --- | --- | --- |
| Enacted PrEP-related stigma | People I care about stopped speaking to me after learning that I take PrEP | 0.747 | 0.777 | 0.642 | 0.806 |
|  | I have lost friends by telling them that I take PrEP | 0.808 | 0.825 | 0.716 | 0.786 |
|  | I have been hurt by how people reacted to learning that I take PrEP | 0.682 | 0.793 | 0.615 | 0.824 |
|  | I regret having told some people that I take PrEP | 0.746 | 0.785 | 0.642 | 0.806 |
|  | I have stopped socialising with some people due to their reaction when learning that I take PrEP | 0.685 | 0.770 | 0.678 | 0.809 |
|  | | | | | |
| Anticipated PrEP-related stigma | Telling someone I take PrEP is risky | 0.757 | 0.851 | 0.789 | 0.860 |
|  | I work hard to keep my PrEP use a secret | 0.884 | 0.839 | 0.790 | 0.866 |
|  | I am very careful whom I tell that I take PrEP | 0.842 | 0.848 | 0.775 | 0.861 |
|  | In many areas of my life, no one knows I take PrEP | 0.843 | 0.804 | 0.709 | 0.870 |
|  | I worry about people discriminating against me because I take PrEP | 0.743 | 0.805 | 0.719 | 0.868 |
|  | I worry that people will assume that because I take PrEP I have sex with lots of people | 0.635 | 0.737 | 0.628 | 0.879 |
|  | I worry that people will assume that because I take PrEP I am HIV positive | 0.428 | 0.578 | 0.436 | 0.901 |

*Based on a two-factor confirmatory factor analysis with oblique rotation to allow for possible correlation between factors. Participants who had not told people that they use PrEP did not respond to the items related to Enacted PrEP-related stigma and were excluded in this sensitivity analysis). Cronbach’s alpha for five Enacted PrEP-related stigma items = 0.839. Cronbach’s alpha for seven Anticipated PrEP-related stigma items = 0.889.

**Supplementary Table S3:** Psychometric properties of PrEP-related stigma scales across the cohort study time points (primary analysis)

| **Scale** | **Time point** | **N** | **Proportion of variance explained by factor** | **Loaded items** | **Cronbach’s alpha** | **Mean (SD)** | **Range** | **% scoring lowest possible score** |
| --- | --- | --- | --- | --- | --- | --- | --- | --- |
| Enacted PrEP-related stigma * | Study entry | 60 | 0.69 | 1 to 5 | 0.85 | 6.8 (2.43) | 5 to 13 | 56.7 |
|  | Follow-up 1 | 57 | 0.69 | 1 to 5 | 0.87 | 6.3 (1.99) | 5 to 11 | 63.2 |
|  | Follow-up 2 | 48 | 0.62 | 1 to 5 | 0.87 | 6.8 (0.20) | 5 to 12 | 53.1 |
|  | Follow-up 3 | 51 | 0.65 | 1 to 5 | 0.91 | 6.5 (2.13) | 5 to 12 | 55.8 |
|  | | | | | | | | |
| Anticipated PrEP-related stigma | Study entry | 60 | 0.24 | 6 to 12 | 0.90 | 14.5 (4.76) | 7 to 28 | 10.0 |
|  | Follow-up 1 | 57 | 0.22 | 6 to 12 | 0.89 | 14.3 (4.63) | 7 to 25 | 3.5 |
|  | Follow-up 2 | 48 | 0.28 | 6 to 12 | 0.88 | 15.2 (4.80) | 7 to 25 | 6.3 |
|  | Follow-up 3 | 51 | 0.26 | 6 to 12 | 0.91 | 14.2 (4.99) | 7 to 25 | 13.7 |

*Participants did not respond to these questions if they had not told others about their PrEP use. Their data have been imputed to strongly disagree (study entry n = 5; follow-up 1 n = 2; follow-up 2 n = 2; follow-up 3 n = 4).

**Supplementary Table S4:** Psychometric properties of PrEP-related stigma scales across the cohort study time points (sensitivity analysis)*

| **Scale** | **Time point** | **N** | **Proportion of variance explained by factor** | **Loaded items** | **Cronbach’s alpha** | **Mean (SD)** | **Range** | **% scoring lowest possible score** |
| --- | --- | --- | --- | --- | --- | --- | --- | --- |
| Enacted PrEP-related stigma | Study entry | 55 | 0.72 | 1 to 5 | 0.84 | 7.0 (2.48) | 5 to 13 | 52.7 |
|  | Follow-up 1 | 55 | 0.72 | 1 to 5 | 0.87 | 6.4 (2.01) | 5 to 11 | 61.8 |
|  | Follow-up 2 | 46 | 0.64 | 1 to 5 | 0.86 | 6.9 (2.23) | 5 to 12 | 50.0 |
|  | Follow-up 3 | 51 | 0.65 | 1 to 5 | 0.90 | 6.5 (2.09) | 5 to 12 | 56.9 |
|  | | | | | | | | |
| Anticipated PrEP-related stigma | Study entry | 55 | 0.20 | 6 to 12 | 0.89 | 14.3 (4.72) | 7 to 28 | 10.9 |
|  | Follow-up 1 | 55 | 0.18 | 6 to 12 | 0.88 | 14.0 (4.45) | 7 to 25 | 3.6 |
|  | Follow-up 2 | 46 | 0.24 | 6 to 11 | 0.87 | 14.8 (4.56) | 7 to 25 | 6.5 |
|  | Follow-up 3 | 51 | 0.27 | 6 to 12 | 0.91 | 14.2 (4.99) | 7 to 25 | 13.7 |

*Participants did not respond to these questions if they had not told others about their PrEP use. Their data have been excluded (study entry n = 5; follow-up 1 n = 2; follow-up 2 n = 2; follow-up 3 n = 4).
